# Walks4work: Rationale and study design to investigate walking at lunchtime in the workplace setting

**DOI:** 10.1186/1471-2458-12-550

**Published:** 2012-07-25

**Authors:** Daniel K Brown, Jo L Barton, Jules Pretty, Valerie F Gladwell

**Affiliations:** 1 School of Biological Sciences, University of Essex, Wivenhoe Park, Colchester, CO4 3SQ, UK

**Keywords:** Walking, Workplace, Physical activity, Cardiovascular disease, Heart rate variability, Nature

## Abstract

**Background:**

Following recruitment of a private sector company, an 8 week lunchtime walking intervention was implemented to examine the effect of the intervention on modifiable cardiovascular disease risk factors, and further to see if walking environment had any further effect on the cardiovascular disease risk factors.

**Methods:**

For phase 1 of the study participants were divided into three groups, two lunchtime walking intervention groups to walk around either an urban or natural environment twice a week during their lunch break over an 8 week period. The third group was a waiting-list control who would be invited to join the walking groups after phase 1. In phase 2 all participants were encouraged to walk during their lunch break on self-selecting routes. Health checks were completed at baseline, end of phase 1 and end of phase 2 in order to measure the impact of the intervention on cardiovascular disease risk. The primary outcome variables of heart rate and heart rate variability were measured to assess autonomic function associated with cardiovascular disease. Secondary outcome variables (Body mass index, blood pressure, fitness, autonomic response to a stressor) related to cardiovascular disease were also measured. The efficacy of the intervention in increasing physical activity was objectively monitored throughout the 8-weeks using an accelerometer device.

**Discussion:**

The results of this study will help in developing interventions with low researcher input with high participant output that may be implemented in the workplace. If effective, this study will highlight the contribution that natural environments can make in the reduction of modifiable cardiovascular disease risk factors within the workplace.

## Background

Non-communicable diseases including cardiovascular disease, diabetes, cancer and chronic respiratory disease caused 36 million deaths world-wide in 2008 [1]. Cardiovascular disease (CVD) is one of the biggest killers in the UK with 193,000 deaths per year (39% of deaths) with huge economic costs (estimated at £30 billion a year in UK) [2]. Furthermore, CVD represents 50% of all European deaths, although 80% of heart disease and stroke incidences are often preventable [3]. Modifiable cardiovascular risk factors include hypertension, hyperlipidemia, physical inactivity, smoking, obesity and diabetes. Some of these modifiable risks can be reduced by increasing physical activity, with a 20-30% greater risk for CVD in those not engaging in regular physical activity [4]. In England, less than 40% of men and 30% of women [5] meet the Government guidelines which are to perform 150 minutes of moderate exercise per week (like brisk walking), or 75 minutes of vigorous physical activity (like jogging or running), or a combination of both in bouts of at least 10 minutes, on a weekly basis [6].

Increasing physical activity does have a range of health benefits but many studies focus on weight loss as a primary outcome. However, a recent article [7] suggests that to determine the beneficial effects of health, body mass should not be the main outcome measure but other markers of health should be assessed including resting and exercising heart rate (HR) and HR recovery (HRR) post-exercise. A slow HRR post-exercise, which is governed at least in part by the autonomic nervous system (ANS) via parasympathetic reactivation, may increase the susceptibility to ventricular fibrillation in patients with ischemic heart disease [8]. HRR post-exercise is also an independent predictor of mortality across large and diverse populations, whether maximal or sub-maximal exercise is performed [9-12]. HRR following exercise may be a better predictor of cardiovascular risk than ECG monitoring, particularly if HRR is combined with cardiorespiratory fitness level another predictor of cardiovascular health [13]. Improvements in cardiorespiratory fitness, irrespective of being considered overweight, can reduce the risk of mortality by all causes and can reduce risk to a greater extent for death from cardiovascular disease [14]. All individuals, therefore, should be encouraged to undertake physical activity [15].

The alterations that are seen in HR by regular physical activity may in part be due to the adaptation of the ANS [16]. ANS control can be studied using heart rate variability (HRV). HRV is a well-established non-invasive tool which gives an indication of the changes in vagal and potentially sympathetic control of the heart [17]. Evidence suggests that low HRV at rest and during exercise may show independent risk for cardiovascular mortality [18]. Reductions in cardiac parasympathetic control i.e. lower baroreceptor sensitivity are highly predictive of cardiovascular mortality [19] and augments the risk of sudden death due to malignant arrhythmias [20,21] particularly in the presence of increased sympathetic drive, as occurs during myocardial ischemia [22]. In previous studies, exercise training has been shown to increase resting HRV, via increases in parasympathetic control of the heart [23]. Exercise training may induce its benefits by altering compliance of the heart and blood vessels, resulting in arterial remodelling of the great vessels, particularly in barosensitive areas and cardiovascular control centres, leading to alterations in parasympathetic outflow [8,24,25].

Low HRV is not only linked to CVD but has also been linked to other diseases including diabetes and depression, and also to stress [26]. The normal response to stress is to induce neuroendocrine changes, which includes the ANS on the neural side, and the hypothalamic-pituitary-adrenal (HPA) axis on the endocrine side. The responses are important as they initiate the fight and flight response, allowing appropriate adaptation to the stress. However, dysregulation of both the ANS and the HPA axis can occur if i) the stress is a major threat, ii) the stressor response is exaggerated, iii) the stress is repetitive and/or iv) recovery following the stressor is attenuated [27]. This can then lead to chronic stress or allostatic load [27]. Alterations in HPA axis can also lead to disturbances in cortisol production, a biochemical marker of stress [28]. Changes in this neuroendocrine response to stressors, with the additional alterations in between behaviours related to stress such as increased smoking, increased alcohol consumption and physical inactivity [29] might explain the association between allostatic load and an increased risk of CVD, specifically coronary heart disease (CHD). Furthermore, stress and lack of time are common factors that are quoted as contributing to reductions in daily physical activity.

Stress is becoming increasingly present in our everyday lives and it is important to explore methods that may help reduce stress, especially as chronic stress can also indirectly cause alterations in behaviours in particular decreased physical activity. Workplace may be a major contributory factor. Cross-sectional studies investigating work stress in men have been associated with repeated activation of the ANS characterised by reductions in HRV [30]. Chronic stress in the workplace is also associated with greater levels in evening salivary cortisol [31]. Consequently, it is important to find methods of encouraging people to become active in an enjoyable way which are sustainable and promote adherence.

Walking is an activity that could be incorporated easily into a working day. Unlike the majority of other physical activities, no specialist equipment is required, apart from maybe a change of shoes and with little need for a shower after the walk. Achieving a certain amount of walking steps per day might significantly contribute to the requirements for moderate physical activity for health benefits [32].

Pedometers have been previously used in interventions not only to measure physical activity by assessing number of steps but also are an effective tool for increasing walking activity in sedentary workers [33]. Number of steps has been related to reductions in resting heart rate and waist circumference when compared to baseline [33] and improvements in quality of life and work performance [34]. The pedometers help to increase general activity throughout the day [35]. In one workplace intervention study, steps/day average increased above pre-intervention levels by 968 steps per day in 179 white collar university workers [36] but no change in sitting time was observed. Although it is difficult to establish an adequate number of walking steps, adults who accumulated at least 10,000 walking steps daily showed decreased body fat and lower BP levels than individuals who did not reach this figure [37]. A limitation of pedometers, however, is at the data retrieval stage, where participants generally need to log activity and data is only collected as a whole day rather than time stamping physical activities throughout the day. Other ways of retrieving physical activity may help to improve the assessment of interventions.

As 50-60% of waking hours are spent at, workplace interventions should be implemented to increase physical activity [38]. Recent recommendations suggest that companies should engage in programmes that improve the health and wellbeing of their employees through specific non-communicable disease prevention schemes and the promotion of healthy lifestyles [38]. Current guidelines suggest that employers should encourage more active transport to and from work, more moving within the working day and promote walking during work breaks [39]. Workplace interventions aimed at changing physical activity and dietary patterns can reduce CVD risk factors such as blood pressure (BP), cholesterol and body mass index (BMI) [40]. Increased levels of walking can also benefit endpoint outcomes (mortality) on CVD [41]. A meta-analysis of workplace physical activity interventions showed participation can have positive effects on fitness, anthropometric measurements as well as work attendance and job stress [42]. A review of 13 intervention trials (8 randomised control and 7 observational studies) concluded that change in physical activity patterns during the working day can also improve psychosocial health [43].

Increasing activity within the working day especially during lunchtime may help to increase overall activity levels. Not only can increases in physical activity and breaks during the day have benefits for the individual but they are deemed to also benefit the employer [44].

An intervention needs to be cost efficient and sustainable in the long-term for them to be effective. This is particularly important in the workplace as companies do not always have large budgets to dedicate to health interventions. Employers, particularly in UK, are not yet aware of the cost to benefit ratio as it more difficult to assess than in the USA where companies pay for the health care of their employees. Further, physical activity interventions in the workplace that have both physiological and psychological benefits, at low cost would be invaluable in reaching a wide working population.

Physical activity levels may be increased by being within nature and/or green space whether it is managed parks, trails or more remote unmanaged environments [45]. Interestingly, is physical activity when combined with nature, it may also have enhanced benefits. Nature may offer reductions in both stress and mental fatigue [46], which would be an additional benefit to individuals and employers during work time. Thus, the combination of nature and exercise during lunchtime may offer a simple and inexpensive solution to increasing physical activity levels and reducing stress [47]. The combination of exercise in nature has been called “green exercise” [48] and the synergistic action of nature and the physical activity may increase the benefits of physical activity. In terms of physiological measures, in a laboratory study, BP was lower 5 minutes post-exercise after viewing images of nature compared to exercising whilst viewing images of built environments [48]. Walking or sitting in a natural (forest) environment has also been shown to lower HR and BP when compared to a built environment control [49]. Park and colleagues suggest that an increase in parasympathetically mediated HRV, with simultaneous decreases in sympathetic components is responsible for the observed reductions in HR and BP [49]. This is supported by the observation that viewing nature alone, within a laboratory, increases parasympathetic activity and overall HR variability [50]. Another study, where walking outdoors in natural environments was used, BP was reduced with a trend to reduced urinary noradrenaline, inferring that this was driven by a decrease in sympathetic stimulation [51]. Prior viewing of nature has also been shown to increase vagal activity following a stressor, suggesting an enhanced recovery (unpublished) and a potential reduction in allostatic load. Nature also impacts on psychological markers of health increasing both mood and self-esteem [52-54], with only 5 minutes of exposure to the natural environment having a large effect [54].

Incorporating “green exercise” into a workplace intervention may have a greater influence on modifiable risk factors for non-communicable diseases, than exercise alone or in built environments. The use of nature as part of a walking intervention in the workplace is a novel approach and the aim of the current study was to investigate whether using “green exercise” as an intervention, improves cardiovascular markers and stress, whilst also enhancing physical and psychological health compared to exercise in a built environment. Furthermore, it was explored whether nature may improve adherence rates.

 To date few studies have used robust data collection methods to measure the impact of workplace interventions on employees’ physical activity levels and health markers [39]. This study was designed to address this by including methods that were both objective and subjective: physical activity monitoring; physical, physiological and psychological markers of health.

In summary, this study posed the following primary research questions:

1. Can walking at lunchtime induce changes in cardiovascular markers that have been previously linked to health?

2. Are the changes modified by the type of walking environment?

Secondary research questions included:

1. What are the effects of walking at lunch time on other markers of health and stress levels?

2. Can a lunch time walking intervention increase overall physical activity (both within and outside work)?

3. Are fitness levels and HRR from exercise and stress altered by walking environment?

4. Does the walking environment affect health outcomes (including stress) and adherence to weekly walks?

5. Do participants have a preference to a particular walk and thus does choice of walk affect adherence?

6. How feasible is it to introduce an 8-week walking intervention to a workplace?

7. At 3 month follow-up what effect is there on health markers?

## Methods

The study is unique as it was also designed to explore the effect of the exercise environment on walking behaviour. A 20 week randomised controlled design with a delayed treatment group was used. The study was designed to assess if a low volume lunchtime walking programme in different environments was effective in terms of alterations in markers of cardiovascular health including HR and markers of vagal activity, specifically HRV. Secondly, it allowed the assessment of whether such interventions were feasible in a private sector company and whether they had an impact on general physical activity, walking behaviour, general and work-related health, specifically stress related measures (including the ability to recover from a stressor).

Randomisation for the grouping of the participants was generated by a computer program. There were three groups initially: a) off-road walking group (NATURE) (n = 32), b) on-road walking (BUILT) (n = 33), c) waiting list control group who joined the treatment group after 8 weeks (n = 29). After the 8 week walking programme all participants (including the waiting list control group) were allowed to undertake any of the walks.

The study was conducted during Spring and Summer 2011.

### Ethical approval

Ethical approval was obtained by NREC Cambridge 4 ethics committee. All participants were treated in accordance with the principles outlined in the Helsinki declaration including gaining informed consent from all participants.

### Recruitment process

Initially, the study was advertised in the University Alumni magazine (Spring 2010) to attract businesses that might be interested. One private sector company responded who did not have an extensive health and well-being programme.

Following informative discussions the company agreed to take part in the Walks4work programme and allow access to their employees (n = 400), mainly scientists and engineers, which were split across several sites. Two sites were selected with n = 161 at one site and n = 217 at the other. Both sites undertook similar work and were both on the outskirts of built environments.

**Figure 1:**
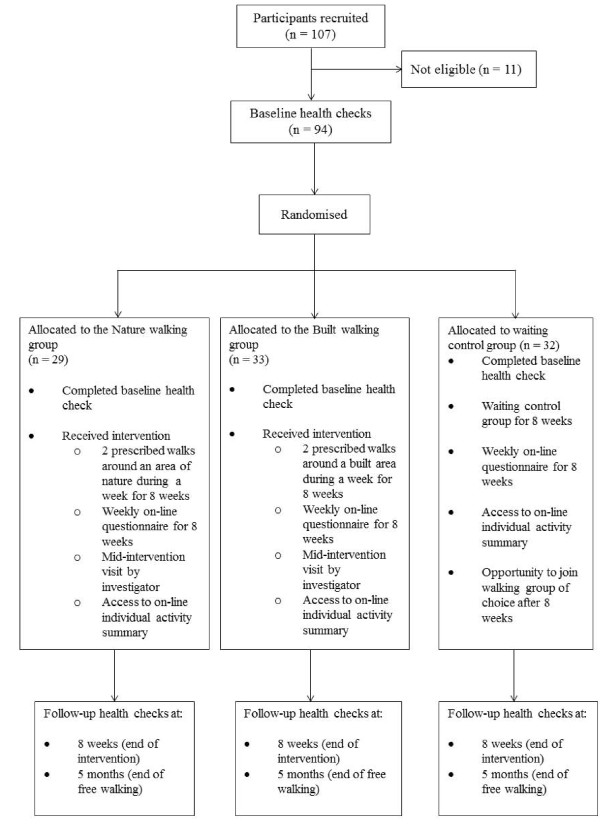
Breakdown of participant recruitment and group allocation at baseline and during the eight week intervention period.

Staff were emailed details about the study by managers, details were also discussed at briefing meetings attended by all staff and on the Managing Director’s blog. On several occasions the researchers attended both sites to talk about the study and to recruit participants that were eligible (see inclusion criteria). A website was also set up to enable prospective participants to gain more information about the study. Prospective participants were also free to email the chief researcher at any time. Posters were placed around the worksites which contained the website address. The flow of participants through the process is presented in Figure 1.

### Power calculation and study population

Change in HR and natural log of high frequency values in HRV spectral analysis (lnHF) are both good markers of health. Improvements in fitness levels are also associated with improved cardiovascular function [55]. Therefore, these parameters were used in the power calculation to inform the number of participants required for the study. Assuming a relevant difference of 0.6 ± 0.5 lnHF and 7.0 ± 7.0 HR, a significance level of α = 0.05, statistical power of 80%, inclusion of three groups and associated Bonferroni adjustments, a final group size of n = 24 was required. However, it was expected that 30% of participants would not comply with the study protocol, which may be due to unusable data (including incomplete data, technical failure), drop-outs and non-finishers due to intervention and/or the follow-up period. Therefore, approximately 32 participants were required per group so we aimed to recruit a total of 100 participants.

Individuals were eligible to take part if they were between 18–65 years old and they considered themselves to be healthy and able to participate in fairly intense exercise. Exclusion criteria excluded individuals who were unable to take part due to cardiovascular and/or neurological conditions or who were taking medication which affects these systems.

### Data collection

Data was collected during a 1 hour visit to a testing room at the worksite (health check). Using Survey Monkey, an on-line questionnaire was completed within two days of testing. Two evening saliva samples (one per evening on two consecutive nights) were taken on or within two days of the health check. This was repeated at three time points: i) baseline, ii) end of Phase 1 (end of 8 week walking intervention) and iii) end of Phase 2 (3 month follow-up).

Participants attended the testing in groups of up to 8. The room was set-up with 2 rows of 4 chairs, which were positioned back to back. This ensured minimal eye contact between participants. Participants completed a consent form and the Pre Exercise Screening Questionnaire, and were then fitted with two HR monitors. A Polar HR monitor (Polar Electro UK Ltd., Warwick, England) was used to enable HR to be viewed during the testing procedure. The other HR recorder (eMotion sensor, Megaemg, University of Kuopio, Finland) was used to assess each heart beat to explore HRV. It was attached by two electrodes (one placed just below the collar bone on the right hand side, the other placed approximately 2 cm above the bottom of the ribs, just to the left of the middle of the sternum). The eMotion sensor did not have a display but recorded each heart beat to facilitate HRV analysis, which was the main outcome measure of the study. Stature (m), body mass (kg) and waist circumference (cm) were also recorded. Participants completed a brief questionnaire comprising a visual analogue scale. They had to mark on a 10 cm line how they felt at that moment in time, using the following descriptors: anxious, tense, stimulated, relaxed, uncomfortable, tired and good. The left hand side of the line (0) represented “not at all” with the right hand side (10) representing “extremely”. A resting BP measurement was taken using an electronic blood pressure monitor (Omron, MX3 basic) with the cuff placed on the participant’s upper right arm, a 5 minute baseline recording of HR was logged. Participants were asked to remain still and quiet with legs uncrossed during the recording period.

The participants then completed a three minute stressor comprising a mental arithmetic challenge. This was undertaken to identify if participants improved following the intervention in their response and recovery to the stressor in terms of HR and HRV. All participants were given a small pad with a sum on the front. It consisted of a four digit number (e.g. 2741) which was followed by a two digit number (13, 17, or 23). The two digit number was subtracted from the four digit number and the answer to this was written on the next page. The same two digit number was then subtracted from the new four digit number. Participants were asked to repeat the process as quickly as possible for a period of 3 minutes. During this time they were instructed only to write the answer and not to write any calculations down or look back at the previous answer. They were told the longest sequence of correct answers would be calculated. To induce a further socio-evaluative threat, participants were watched carefully by the researchers who walked around the room looking at the answers provided. A count down was given at half-way, one minute to go, 30 seconds remaining and 10 seconds left to increase the pressure on the participants. At the end participants were asked to place their pen and paper down and to remain seated with legs uncrossed with no talking for a further 3 minutes to allow recovery heart rate to be recorded. BP was measured using the standardised procedure described earlier and the same visual analogue scale was also completed.

The final stage of the health check was to measure fitness levels using a Chester Step test. This is a sub-maximal test that predicts maximal oxygen uptake based on HR recorded at the end of each stage up to 80% of their maximum HR (which is based on a maximum of 220-age). The data is then extrapolated to their maximum HR. It has good reliability and validity [56,57]. The step test involves a maximum of five 2 minute stages of increasing stepping frequency in time to a beat played via a CD player. Participants were asked to step until they reached about 80% of their maximum heart rate (previously calculated by researchers) or a rate of perceived exertion of about 14 on the 6–20 Borg scale [58]. Step height was determined based on age and physical activity levels: Low step (15 cm)- over 40 years old and low physical activity levels; Medium step (20 cm)- over 40 years old but higher levels of physical activity or under 40 and low levels of physical activity; High Step (25 cm)-under 40 and physically active.

HR was recorded throughout the fitness test with both Polar and eMotion monitors. The heart rate was noted from the Polar monitor at each stage, as was rate of perceived exertion. After participants reached 80% of their maximum HR they were asked to sit down, relax and not talk. HR was noted at every minute during the recovery period from the Polar heart rate monitor to allow HRR to be determined. BP was then measured and recorded 3 minutes after the end of their stepping task.

Questionnaires were completed on-line (Survey Monkey). No-one requested a paper based copy, although this was available if required. The questionnaires were completed within five days of each of the health checks. The questions included: physical activity levels (IPAQ) [59], mood (PANAS) [60], self-esteem (Rosenberg) [61], health (SF-8) ( http://www.sf-36.org (Medical Outcomes Trust)), perceived stress (PSS) [62], job satisfaction (Utretch workplace engagement scale) [63,64]. At the end of Phase 2 (3 month follow-up) nature relatedness was also completed [65]. This was used as a trait measure to find out how participants related to the environment.

Additionally, during the 8 week intervention period, participants were asked to complete a weekly on-line questionnaire. This was to track participant’s subjective activity levels (IPAQ), mood (PANAS), self-esteem and perceived stress [66] during the intervention.

### Physical activity monitoring

Physical activity was monitored in three ways: diary, questionnaire (IPAQ as above) and by using an objective measure, an ActiPed activity monitor.

#### IPAQ

The short form of the International Physical activity questionnaire was used. This asked questions about number of days per week and time per day on average spent doing vigorous and moderate (excluding walking) activities. Walking was included as a separate category. Average sitting time per day was also included.

#### Diaries

In Phase 1, participants were asked to complete a diary which included all the physical activity they did during the week, comprising type, duration and intensity. This enabled comparison to other methods of assessing physical activity.

In Phase 2, participants were asked to complete an on-line diary mainly about their lunch time activity, chosen type of walking environment and whether it was one of the intervention walks.

#### Activity monitor

The ActiPed (FitLinxx Inc., Shelton, CT, USA) (also known as LifeSource XL-20 wireless activity monitor) evaluates the number of steps in activities such as walking and running. It is an unobtrusive, small, and light instrument. It counts steps by measuring the acceleration of the foot and processes this information to determine the time of foot contact on the ground and the time of the swing phase [67,68]. Furthermore, it provides a simple output of number of steps, distance travelled, time of activity and energy expenditure by sending the data wirelessly through integrated radio frequency to an ActiLink, which is a USB Human Interface Device receiver attached to an internet-linked computer. In large scale studies the data from many ActiPeds can be uploaded to an ActiLink on a single computer and the information is then available for use via a secure individual webpage for each of the participants. Furthermore, the researchers can also view all of the data for each participant and can also log into each of the individual webpages of the participants. The ActiPed has no display, and data recorded can only be viewed on-line.

### Groupings for physical activity intervention

There were 3 groups in total: waiting control (n = 29), built walk (n = 33) and nature walk (or off-road as it was known to participants) (n = 32). Groups were randomly assigned using a random number generator prior to attending the first health check which accounts for the slight variation in distribution of numbers. Grouping information was not released to participants or researchers until after the baseline health check.

### The intervention

Participants who were in a walking group were instructed to walk their allocated route twice per week. The walk was approximately 2 km and took around 20 minutes to complete (equating to approximately 2000 steps). Participants were supplied with a map of the walk with instructions including pictures of the route. Prior to the intervention period, participants in the different walking groups were invited by the researchers to attend a practice walk session where they were shown the route. It was suggested that they walk one way round for one of the weekly walks, and the other way for the second walk.

Participants were asked to continue to wear their ActiPed monitor throughout the walk and to also wear a Polar heart rate monitor. They were asked to walk at 60% of their HR maximum to ensure they walked at a moderate pace. They were also asked to be guided by how they were feeling and to walk at an intensity of 12 on the Rate of Perceived exertion scale (Borg 6–20).

#### Phase 2

In Phase 2, all participants were encouraged to walk one of the walks that had been designed for the study however there was no encouragement to do a particular walk. This was in order to try not to bias which walk the participants chose as one of the secondary outcome measures was to identify which walk was most preferred.

### Outcome measures

The primary outcome measure was HR and lnHF (to determine adaptations in autonomic control specifically vagal autonomic control). This was assessed at each health check to provide baseline data, during and in recovery from a stressor and exercise test. Secondary outcome measures included stress, health and physical activity. Stress was measured using perceived stress and also objectively using evening salivary cortisol. Markers of health including fitness, BMI, SF-8, mood and self-esteem as well as job satisfaction were measured. Physical activity was measured objectively using activity monitors (ActiPed) and subjectively by questionnaire (IPAQ) and diary. Nature relatedness was assessed at the end of the study and used as a trait measure to find out how participants related to the environment.

### Statistical analysis

Initially the effect of the walking intervention (irrespective of walking environment) will be analysed to explore whether walking at lunchtime induced significant changes in cardiovascular markers (research question 1). The data from the end of the 8 week intervention (phase 1) will be analysed using a mixed ANOVA using time (base and end phase 1) and group. A Bonferroni correction will be applied where appropriate. To further examine if the changes were modified by the walking environment (research question 2) again a mixed ANOVA will be used and the differences between the 3 groups will be explored. For secondary research questions including whether the Walks4work programme can increase general physical activity, fitness levels, HRR from stressor and exercise test and also lead to decreases in objective and perceived markers of stress again mixed ANOVA will be used. Post-hoc tests will be applied as appropriate. Multilevel modelling will be used to examine how within-person changes in the predictor variables (physical activity and number of lunchtime walks) can predict within-person changes in the outcome variables (including health markers and stress). Data will be analysed in this way for both Phase 1 and Phase 2 and will also take into account walking environment. To assess whether a walking intervention in a workplace is feasible adherence to walks will be assessed both objectively (using ActiPed data) and subjectively from diaries.

## Discussion

This paper describes the background and methodology of a study designed to investigate cardiovascular markers of health following a workplace walking intervention. Participants walked twice weekly over an 8 week period with a 3 month follow-up to establish adherence. Participants were randomly allocated to different walking environments and environmental preferences were also assessed. The study used a randomised controlled design with a delayed treatment control group to examine the effect of the proposed lunchtime walking intervention on key outcomes and also secondary outcomes. Previous workplace interventions are limited as they have mainly been conducted in a University environment which is not a true representation of workplaces. This project aimed to target a whole workplace across all levels of employment including the management team. One of the strengths of this study is that the outcomes were both objectively and subjectively measured, which has been used as a criticism of previous studies [39]. This included objectively measured physical activity using innovative technology which did not rely on participants noting down the number of steps they did in a day, which is the usual practice for pedometer studies. Furthermore, stress was also measured objectively using evening cortisol levels. Evening cortisol salivary levels have been shown to give an indication of chronic stress whereas the morning awakening response is more reactive to daily stress [31]. The relationship between objective and subjective measures will be also explored which has important implications as they do not always have a linear relation.

A walking intervention is well suited to both a workplace and less active individuals. A lunchtime walking programme, also targets those employees who struggle to fit in sufficient levels of physical activity outside of work but may find that the workplace offers an ideal place to increase physical activity and to adhere to a physically active lifestyle. Although one of the limitations of this study was that participants were only asked to walk twice per week for 25 minutes which is below the recommendations for physical activity for health [69]. To meet the physical activity guidelines, participants would need to walk every day during lunchtime, but when the population has previously been sedentary, this is likely to have low compliance, especially if a walk leader is not present. To date, the effect of low frequency walking prescription on health markers in particular cardiovascular control and stress are not well investigated. One previous study examined 8 weeks walking twice per week for 45 minutes and found significant reductions in systolic BP [70]. However, it is hoped that the walks4work programme will enable participants to find something they enjoy that fits into their daily life and also be a facilitator for overall change in incorporating physical activity into their lives.

Previously it has been shown that there may be a synergistic effect of exercise combined with nature (“green exercise”) but to our knowledge, this is the first study to explore the use of the environment in a workplace physical activity intervention. The aim of the study was to investigate whether walking in nature had any enhanced benefits over walking in a built environment. It is hoped that the intervention will reap benefit for employees and employers. If the intervention proves successful, employee health and well-being can be enhanced including cardiovascular risk and a reduction in stress.

The results of the intervention (including the three month follow-up) are expected to be analysed by May 2012.

## Abbreviations

ACSM: American College of Sports Medicine; ANOVA: Analysis of Variance; ANS:Autonomic Nervous system Control; BASES: British Association of Sport and Exercise Science; BMI Body: Mass Index; BP: Blood pressure; CHD: Coronary heart disease; CVD: Cardiovascular disease; HPA: Hypothalamic-pituitary-adrenal; HR: Heart rate; HRR: Heart rate recovery; HRV: Heart rate variability; IPAQ: International Physical Activity Questionnaire; lnHF: Natural logarithm of high frequency; NICE: National Institute for Health and Clinical Excellence; NREC: National research ethics committee; PSS: perceived stress scale; WHO: World Health Organisation.

## Competing interests

The authors declare that they have no competing interests.

## Author’s contribution

DB, JB, JP, VG designed the study and wrote the initial protocol. DB, JB and VG collected data. DB and VG analysed data. VG and DB delivered elements of the intervention. VG and DB co-ordinated the study. VG and DB drafted the manuscript. All authors read, provided comments and approved the final manuscript.

## Authors information

School of Biological Sciences, University of Essex. Colchester. CO4 3SQ.

## Pre-publication history

The pre-publication history for this paper can be accessed here:

http://www.biomedcentral.com/1471-2458/12/550/prepub
